# Effect of SARS-CoV-2 BNT162b2 mRNA vaccine on thyroid autoimmunity: A twelve-month follow-up study

**DOI:** 10.3389/fendo.2023.1058007

**Published:** 2023-01-27

**Authors:** Shuhei Morita, Tomoyuki Takagi, Hidefumi Inaba, Yasushi Furukawa, Shohei Kishimoto, Shinsuke Uraki, Naoki Shimo, Ken Takeshima, Saya Uraki, Kei Doi, Mitsuyo Imagawa, Mika Kokawa, Tomomi Konami, Hitomi Hara, Yoshihiro Hara, Emiko Sone, Hiroto Furuta, Masahiro Nishi, Asako Doi, Shinobu Tamura, Taka-aki Matsuoka

**Affiliations:** ^1^ First Department of Medicine, Wakayama Medical University, Wakayama, Japan; ^2^ Wakayama City Medical Association Seijinbyo Center, Wakayama, Japan; ^3^ Department of Diabetes and Endocrinology, Japanese Red Cross Wakayama Medical Center, Wakayama, Japan; ^4^ Department of Emergency and Critical Care Medicine, Wakayama Medical University, Wakayama, Japan

**Keywords:** SARS-CoV-2, COVID-19, thyroid autoimmunity, BNT162b2 vaccine, thyroid-stimulating hormone receptor antibody, Graves’ disease

## Abstract

**Objectives:**

Graves’ disease (GD) has been highlighted as a possible adverse effect of the respiratory syndrome coronavirus-2 (SARS-CoV-2) vaccine. However, it is unknown if the SARS-CoV-2 vaccine disrupts thyroid autoimmunity. We aimed to present long-term follow-up of thyroid autoimmunity after the SARS-CoV-2 BNT162b2 mRNA vaccine.

**Methods:**

Serum samples collected from seventy Japanese healthcare workers at baseline, 32 weeks after the second dose (pre-third dose), and 4 weeks after the third dose of the vaccine were analyzed. The time courses of anti-SARS-CoV-2 spike immunoglobulin G (IgG) antibody, thyroid-stimulating hormone receptor antibody (TRAb), and thyroid function were evaluated. Anti-thyroglobulin antibodies (TgAb) and anti-thyroid peroxidase antibodies (TPOAb) were additionally evaluated in thirty-three participants.

**Results:**

The median age was 50 (IQR, 38-54) years and 69% were female. The median anti-spike IgG antibody titer was 17627 (IQR, 10898-24175) U/mL 4 weeks after the third dose. The mean TRAb was significantly increased from 0.81 (SD, 0.05) IU/L at baseline to 0.97 (SD, 0.30) IU/L 4 weeks after the third dose without functional changes. An increase in TRAb was positively associated with female sex (β = 0.32, *P* = 0.008) and low basal FT4 (β = -0.29, *P* = 0.02) and FT3 (β = -0.33, *P* = 0.004). TgAb was increased by the third dose. Increase in TgAb was associated with history of the thyroid diseases (β = 0.55, *P <*0.001).

**Conclusions:**

SARS-CoV-2 BNT162b2 mRNA vaccine can disrupt thyroid autoimmunity. Clinicians should consider the possibility that the SARS-CoV-2 vaccine may disrupt thyroid autoimmunity.

## Introduction

For the COVID-19 pandemic, the severe acute respiratory syndrome coronavirus 2 (SARS-CoV-2) mRNA vaccines have been authorized and have provided durable benefits in reducing the risk of the severe outcomes of hospitalization and death (1, 2). To date (May 2022), three doses of SARS-CoV-2 mRNA vaccination (the initial two doses and the third ‘booster’ dose) have been introduced in the many countries, including Japan. As the fourth doses of the SARS-CoV-2 mRNA vaccines were only authorized for those older than 50 years in the US, the vaccination program is speculated to be continued specifically in the population who are at greatest risk and who might gain most benefit from vaccination. This includes immunocompromised individuals and people older than 50 years, given the prevalence of comorbidities that increase the risk of severe disease and death in such individuals (1, 2).

Despite the robust beneficial data on the SARS-CoV-2 mRNA vaccine, adverse effects of the vaccines related to endocrine disorders have also recently been highlighted (3–8). Among the endocrine organs possibly targeted as adverse effects of the vaccine, the thyroid gland is the most common one (4, 7). Notably, an increasing number of cases of new-onset or relapse of Graves’ disease (GD) has been recently reported to occur following the SARS-CoV-2 mRNA vaccines (3, 4, 7, 9). Such case reports and series have raised speculation of the development of thyroid autoimmunity due to the mRNA vaccine. However, there is limited epidemiological evidence or basic findings to clarify if the SARS-CoV-2 mRNA vaccine induces thyroid autoimmunity. Furthermore, if the mRNA vaccine disrupts thyroid autoimmunity, further clinically important questions arise: if the disruption has the potential to induce functionally overt situations, how long does the vaccine affect thyroid autoimmunity, and what factors predict its disruption?

In this study, we present a twelve-month clinical follow-up of thyroid-stimulating hormone (TSH) receptor antibody (TRAb) as a marker of thyroid autoimmunity disease, GD, and thyroid function from the baseline to after the third dose of the SARS-CoV-2 BNT162b2 (Pfizer/BioNTech) mRNA vaccine in Japanese healthcare workers. In addition, if induction of TRAb could be observed after the vaccine, we aimed to investigate factors that could be used to predict it.

## Materials and methods

### Study design and timeline

**Figure 1:**
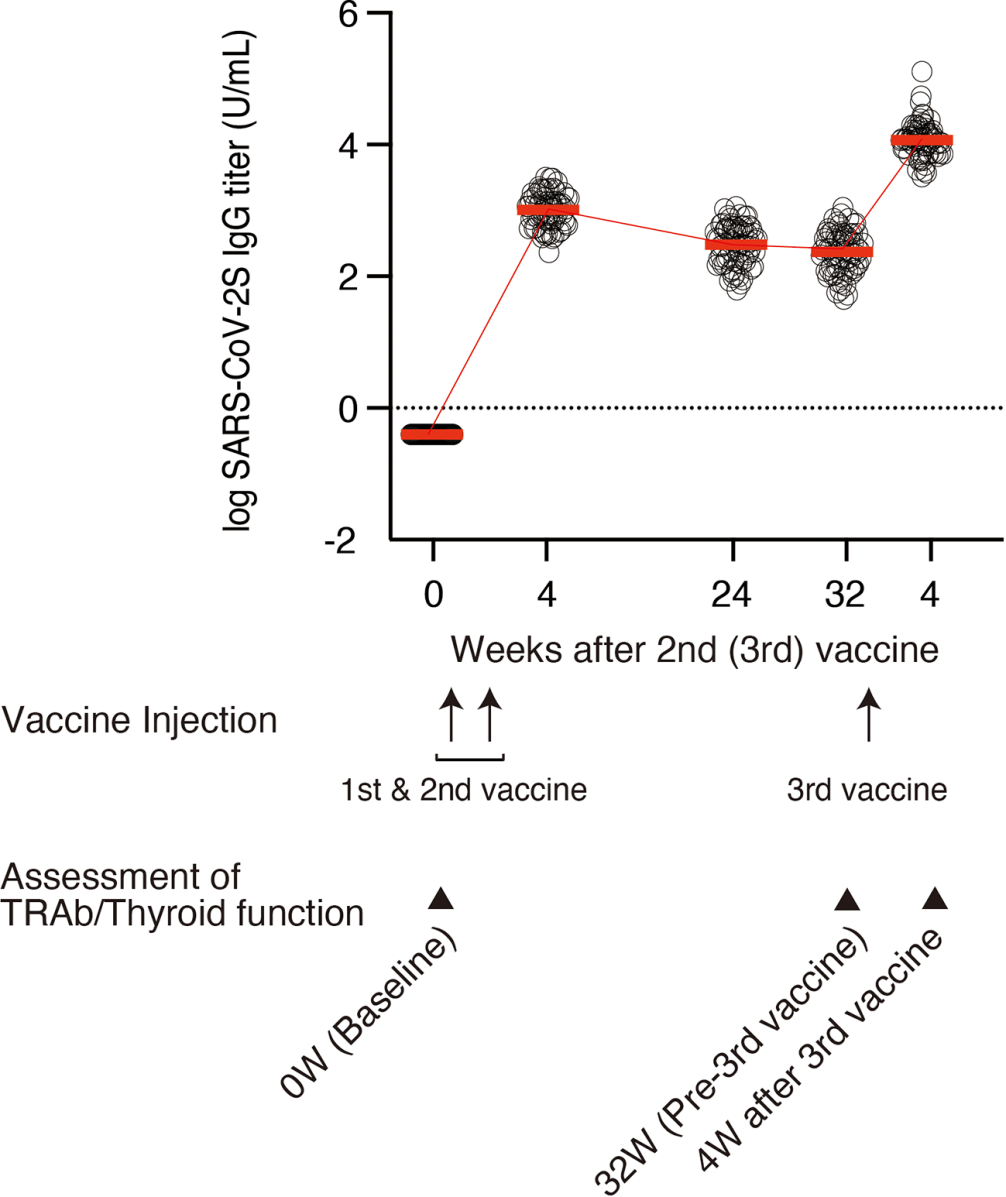
Timelines and trends of logarithmically transformed anti-SARS-CoV-2 S IgG titers at 0 (baseline), 4, 24, and 32 weeks after the second dose of vaccine and 4 weeks after the third dose of vaccine (n = 70). Each circle denotes individual log IgG titers. Mean values of anti-SARS-CoV-2 S IgG titer are indicated by red bars. Mean values are also presented in Table 1. TRAb and thyroid function were analyzed at 0 (baseline), 32 weeks after the second dose, and 4 weeks after the third dose of vaccine. TgAb and TPOAb were also assessed at 32 weeks after the second dose and 4 weeks after the third dose of vaccine in 33 participants.

In this study, 99 healthcare workers, all employees of Wakayama City Medical Association, were enrolled. The initial interviews and the first serum sampling for the baseline were conducted in March and April 2021. Negativity for SARS-CoV-2 antibodies was confirmed at baseline. Participants received first and second doses of the BNT162b2 mRNA vaccine 3 weeks apart in April and May 2021, and a third dose in January 2022. Serum samples were prospectively collected at 4, 24, and 32 weeks after the second dose, and 4 weeks after the third dose in March 2022 as shown on the time schedule in **Figure 1**. Anti-SARS-CoV-2 antibody titers were measured at each time point. The remaining serum samples were preserved at -80°C until the assessment of thyroid autoimmunity and function. Participants who had consistently elevated titers were evaluated for infection *via* anti-nucleocapsid antibodies.

Exclusion criteria were as follows: i) pregnant and breastfeeding females, ii) thyroid cancer, iii) individuals with serious medical diseases including liver or kidney dysfunction, iv) individuals with breakthrough infections of COVID-19, and v) non-completion of the time schedule. Regarding criterion iii), all the participants were assessed by both medical interview and laboratory test at baseline, especially for liver and renal function, including blood cell counts, glutamic oxaloacetic transaminase, glutamic pyruvic transaminase, γ-glutamyl transpeptidase, creatinine, and creatinine-based estimated glomerular filtration rate.

Among the 99 participants enrolled, several participants were excluded from analysis: three contracted breakthrough infections, five did not receive a third dose of the vaccine, and one was treated for GD with positive TRAb (3.7 IU/L) at baseline were excluded from the analysis. Among the 90 remaining participants, samples from 70 participants whose serum samples at all the time points were sufficient for measurement of thyroid autoimmune antibodies and functions were retrospectively analyzed (**Figure S1**). None of these 70 participants met the exclusion criteria. In a limited number of the participants (n = 33), anti-thyroglobulin antibodies (TgAb) and anti-thyroid peroxidase antibodies (TPOAb) were also analyzed at 32 weeks after the second dose (pre-third dose) and 4 weeks after the third dose (post-third dose) to assess the response of these antibodies to the third dose.

This study was approved by the ethics committee of Wakayama City Medical Association Seijinbyo Center Ethics Committee (No. 202103-1). All participants provided written informed consent.

### Measurement of Anti-SARS-CoV-2 S antibodies

Elecsys Anti-SARS-CoV-2 S immunoassay (Roche Diagnostics, Basel, Switzerland) was used to measure anti-spike immunoglobulin G (IgG) SARS-CoV-2 antibody titers on a Roche Cobas e411 analyzer (Roche Diagnostics) according to the manufacturer’s instructions. Samples with a titer >250 U/mL were serially diluted until the titer became ≤250 U/mL, according to the manufacturer’s protocol. Anti-nucleocapsid antibodies (Elecsys Anti-SARS-CoV-2, Roche Diagnostics) were measured according to the manufacturer’s protocol.

### Measurement of thyroid autoimmune antibodies and functions

TRAb (Elecsys Anti-TRAb v2, Roche Diagnostics; reference range, <2.0 IU/L), TgAb (Elecsys Anti-Tg, Roche Diagnostics; reference range, <28.0 IU/mL), and TPOAb (Elecsys Anti-TPO, Roche Diagnostics; reference range, <16.0 IU/mL) were measured on a Roche Cobas e801 analyzer (Roche Diagnostics) according to the manufacturer’s instructions. TSH (Elecsys TSH v2, Roche Diagnostics; reference range, 0.500-5.000 μIU/mL), free T4 (FT4, Elecsys FT4III, Roche Diagnostics; reference range, 0.90-1.7 ng/dL), and free T3 (FT3, Elecsys FT3III, Roche Diagnostics; reference range, 2.30-4.00 pg/mL) were measured on a Roche Cobas e801 analyzer (Roche Diagnostics) according to the manufacturer’s instructions. Increases of TRAb, TSH, FT4, and FT3 from baseline to 4 weeks after the third dose were defined as ΔTRAb, ΔTSH, ΔFT4, and ΔFT3, respectively, which were calculated as:

(value at 4 weeks after the third dose) - (value at baseline).

Increases of TgAb and TPOAb from 32 weeks after the second dose (pre-third dose) to 4 weeks after the third dose (post-third dose) were also defined as ΔTgAb and ΔTPOAb, respectively, and calculated as:

(vales at 4 weeks after the third dose) - (values at 32 weeks after the second dose).

Responders to increase in TRAb were defined as the subjects who exhibited increase in TRAb in a time dependent manner and had TRAb >1.2 IU/L at 4 weeks after the third dose.

### Statistical analysis

The differences in TRAb, TSH, FT4, and FT3 of each group at each pair of time-points were analyzed using Kruskal-Wallis test followed by Dunn’s multiple comparisons test. The differences in TgAb and TPOAb between 32 weeks after the second dose (pre-third dose) and 4 weeks after the third dose (post-third dose) were analyzed by Wilcoxon matched-pairs signed-rank test. To identify the predictive factors of increase TRAb and TgAb, the associations between ΔTRAb or ΔTgAb and baseline characteristics were analyzed using univariate linear regression. Similarly, the associations between TRAb or TgAb at 4 weeks after the third vaccine and baseline characteristics were also analyzed using univariate linear regression. Differences between responders and non-responders were analyzed using Mann-Whitney U test. A two-sided *P* < 0.05 was considered significant. Statistical analysis was performed using GraphPad Prism version 9.1.0 (GraphPad Software, San Diego, CA, USA) and JMP Pro16 (SAS Inc., Cary, NC, USA).

## Results

### Clinical characteristics and time course of anti-SARS-CoV-2 S antibodies

Clinical characteristics of the 70 participants are shown in **Table 1**. The median age was 50 (interquartile range [IQR], 38-54) years and 69% were female. The time course of anti-SARS-CoV-2 S antibody titer is shown in **Table 1** and **Figure 1**. During the period between 32 weeks after the administration of the second dose and 4 weeks after the third dose, the median antibody titer increased from 325 (IQR, 169-546) U/mL to 17627 (IQR, 10898-24175) U/mL (**Table 1** and **Figure 1**). The period from 32 weeks after the administration of the second dose to 4 weeks after the third dose was 91 (IQR, 91-93) days. All participants had an increase in the antibody titer (**Figure 1**). No major adverse events were reported.

**Table 1:** Baseline cohort characteristics and time course of Anti-SARS-CoV-2 S IgG antibody, TRAb, TSH, FT4, and FT3 (n = 70).

Characteristics	Value
Age (y), median (IQR)	50 (38 – 54)
Sex (Female), n (%)	48 (69)
Body mass index (kg/m^2^), median (IQR)	22 (20 – 24)
Smoking, n (%)	14 (20)
Alcohol (g/week), median (IQR)	0 (0 – 31)
Fever, n (%)	23 (33)
History of thyroid disease, n (%)	4 (6)
Family history of thyroid disease, n (%)	3 (4)
Comorbidities	
Asthma, n (%)	3 (4)
Hypertension, n (%)	9 (13)
Dyslipidemia, n (%)	8 (11)
Malignancy, n (%)	2 (3)
Diabetes mellitus, n (%)	0 (0)
Autoimmune disease, n (%)	2 (3)
Cerebral infarction, n (%)	1 (1)
Current medication	
Allergy, n (%)	9 (13)
Hypertension, n (%)	8 (11)
Dyslipidemia, n (%)	8 (11)
Diabetes mellitus, n (%)	0 (0)
Immunosuppressant, n (%)	2 (3)
Anti-SARS-CoV-2 S IgG antibody	
Baseline IgG titer (U/mL), median (IQR)	<0.4 (<0.4-<0.4)
4 weeks after vaccination	
Days after 2nd dose, median (IQR)	28 (28-28)
IgG titer (U/mL), median (IQR)	1158 (717-1611)
24 weeks after vaccination	
Days after 2nd dose, median (IQR)	168 (168-168)
IgG titer (U/mL), median (IQR)	417 (223-712)
32 weeks after vaccination	
Days after 2nd dose, median (IQR)	224 (224-224)
IgG titer (U/mL), median (IQR)	325 (169-546)
4 weeks after 3rd vaccination	
Days after 3rd dose, median (IQR)	28 (28-28)
IgG titer (U/mL), median (IQR)	17627 (10898-24175)
TRAb (IU/L)	
Baseline, mean (SD)	0.81 (0.05)
32w, mean (SD)	0.91 (0.15)
4 weeks after the 3rd dose, mean (SD)	0.97 (0.30)
TSH (μIU/mL)	
Baseline, mean (SD)	2.06 (1.89)
32w, mean (SD)	1.95 (1.36)
4 weeks after the 3rd dose, mean (SD)	1.72 (1.13)
FT4 (ng/mL)	
Baseline, mean (SD)	1.21 (0.15)
32w, mean (SD)	1.22 (0.16)
4 weeks after the 3rd dose, mean (SD)	1.19 (0.17)
FT3 (pg/mL)	
Baseline, mean (SD)	2.98 (0.41)
32w, mean (SD)	3.08 (0.36)
4 weeks after the 3rd dose, mean (SD)	2.93 (0.41)

IQR, interquartile range.

Sex was recorded as 0: male and 1: female.

### Time course of TRAb and thyroid function

**Figure 2:**
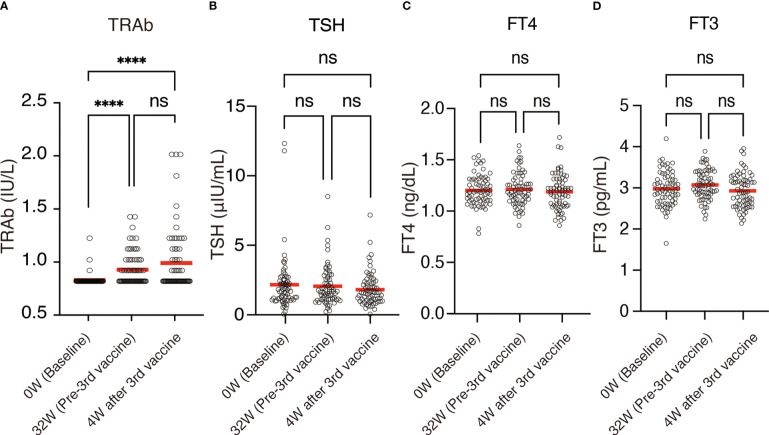
Time course of **(A)** TRAb, **(B)** TSH, **(C)** FT4, and **(D)** FT3 (n = 70). Each circle denotes individual values. Mean values were indicated by red bars. Mean values are also presented in **Table 1**. ns, not significant; ****P <0.0001.

**Figure 3:**
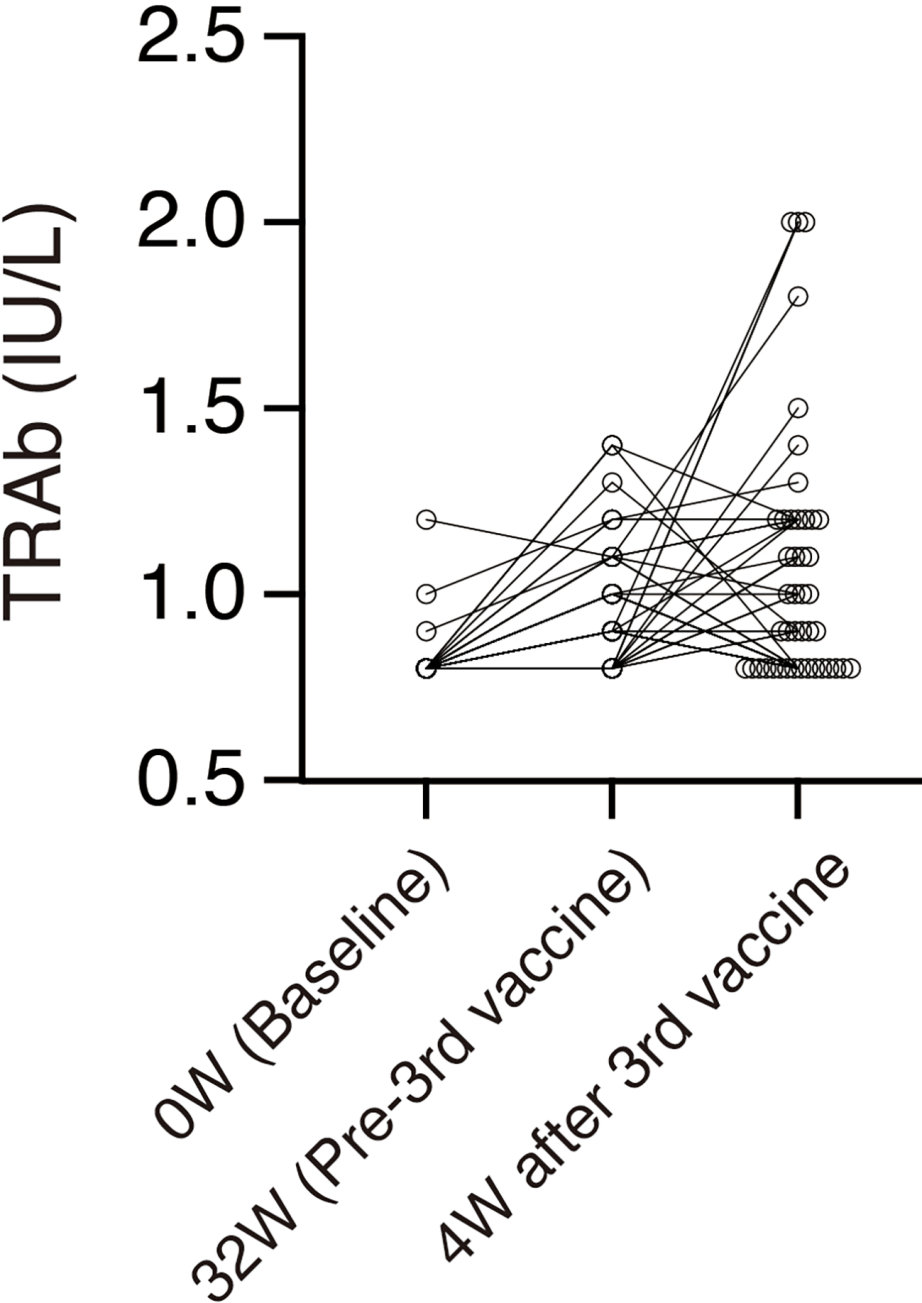
Time courses of TRAb in individuals whose TRAb is more than the lower limit of the range (0.8 IU/L) at any time point. Each circle denotes an individual value. n = 44.

The mean TRAb increased from 0.81 (SD, 0.05) IU/L at baseline to 0.91 (SD, 0.15) IU/L 32 weeks after the second dose (*P <*0.0001) and 0.97 (SD, 0.30) IU/L 4 weeks after the third dose (*P <*0.0001) (**Figures 2A**, **3**, **Table 1**). On the other hand, thyroid functions assessed by TSH, FT4, and FT3 were not significantly changed between each time points, although TSH showed a trend of time-dependently decrease following the mRNA vaccine (**Figures 2B–D**, **Table 1**). No participants showed definite symptoms of hyperthyroidism at any time point.

### Predictive factors for the increase of TRAb

To investigate the factors which could contribute to the increase of TRAb, we first calculated ΔTRAb as described in the Materials and Methods section. Univariate analysis for ΔTRAb was then performed to identify the predictive variants at baseline. ΔTRAb was positively associated with female sex (β = 0.315, *P* = 0.008), low basal FT4 (β = -0.285, *P* = 0.02), and low basal FT3 (β = -0.333, *P* = 0.004) (**Table 2**). As confirmation, similar findings were also observed by univariate analysis for TRAb 4 weeks after the third dose (**Table 2**).

**Table 2:** Univariate analysis for increases of TRAb (ΔTRAb) and TRAb 4 weeks after the third dose vaccine (n = 70).

	ΔTRAb		TRAb (4 weeks after the third dose)	
Variable	β (95% CI)	*P value*	β (95% CI)	* P value*
Age (y)	-0.19 (-0.41 to 0.05)	.11	-0.14 (-0.36 to 0.10)	.24
Sex	0.32 (0.09 to 0.51)	.01	0.31 (0.08 to 0.51)	.01
Body mass index (kg/m^2^)	-0.04 (-0.27 to 0.20)	.75	-0.05 (-0.28 to 0.19)	.70
Smoking	-0.08 (-0.31 to 0.16)	.52	-0.08 (-0.31 to 0.16)	.51
Alcohol (g/week)	-0.04 (-0.27 to 0.20)	.74	-0.01 (-0.25 to 0.22)	.92
Fever	0.21 (-0.03 to 0.42)	.08	0.18 (-0.06 to 0.40)	.14
History of thyroid disease	0.15 (-0.09 to 0.37)	.23	0.13 (-0.11 to 0.35)	.28
Family history of thyroid disease	0.03 (-0.21 to 0.26)	.80	0.02 (-0.21 to 0.26)	.85
**Comorbidities**				
Asthma	-0.02 (-0.25 to 0.22)	.88	-0.03 (-0.26 to 0.21)	.84
Hypertension	-0.08 (-0.31 to 0.16)	.51	-0.03 (-0.26 to 0.21)	.80
Dyslipidemia	-0.03 (-0.26 to 0.21)	.83	0.02 (-0.21 to 0.26)	.85
Thyroid disease	0.15 (-0.09 to 0.37)	.23	0.13 (-0.11 to 0.35)	.28
Malignancy	0.15 (-0.09 to 0.37)	.23	0.13 (-0.10 to 0.36)	.27
Autoimmune disease	0.09 (-0.15 to 0.31)	.48	0.08 (-0.16 to 0.31)	.53
Cerebral infarction	-0.07 (-0.30 to 0.17)	.58	-0.07 (-0.30 to 0.17)	.57
Current medication				
Allergy	-0.11 (-0.34 to 0.13)	.36	-0.12 (-0.34 to 0.12)	.33
Hypertension	-0.06 (-0.29 to 0.18)	.63	-0.01 (-0.24 to 0.23)	.95
Dyslipidemia	-0.03 (-0.26 to 0.21)	.83	0.02 (-0.21 to 0.26)	.85
Thyroid disease	0.08 (-0.16 to 0.31)	.50	0.07 (-0.17 to 0.30)	.56
Immunosuppressant	0.09 (-0.15 to 0.31)	.48	0.08 (-0.16 to 0.31)	.53
**Anti-SARS-CoV-2 S Ab IgG titer (U/mL)**				
Pre-vaccination (Baseline)	N/A		N/A	
4 weeks after the 2nd dose	-0.05 (-0.28 to 0.19)	.68	-0.07 (-0.30 to 0.17)	.55
24 weeks after the 2nd dose	0.10 (-0.14 to 0.32)	.43	0.06 (-0.18 to 0.29)	.62
32 weeks after the 2nd dose	0.04 (-0.20 to 0.27)	.76	0.01 (-0.23 to 0.24)	.95
4 weeks after the 3rd dose	-0.09 (-0.31 to 0.15)	.48	-0.09 (-0.32 to 0.15)	.45
**TRAb (IU/L)**				
Baseline	0.17 (-0.07 to 0.39)	.16	0.34 (0.12 to 0.53)	.00
32w	0.09 (-0.14 to 0.32)	.44	0.14 (-0.10 to 0.36)	.25
4 weeks after the 3rd dose	0.98 (0.97 to 0.99)	.00	N/A	
ΔTRAb	N/A		0.98 (0.97 to 0.99)	.00
TSH (μIU/mL)				
Baseline	-0.06 (-0.29 to 0.18)	.64	-0.04 (-0.27 to 0.19)	.73
32w	-0.14 (-0.36 to 0.10)	.26	-0.09 (-0.32 to 0.15)	.45
4 weeks after the 3rd dose	-0.19 (-0.40 to 0.05)	.12	-0.15 (-0.38 to 0.08)	.20
ΔTSH	-0.07 (-0.30 to 0.17)	.56	-0.07 (-0.30 to 0.17)	.59
FT4 (ng/dL)				
Baseline	-0.29 (-0.49 to -0.05)	.02	-0.29 (-0.49 to -0.06)	.02
32w	-0.16 (-0.38 to 0.08)	.18	-0.16 (-0.38 to 0.07)	.17
4 weeks after the 3rd dose	-0.09 (-0.32 to 0.15)	.46	-0.11 (-0.34 to 0.13)	.37
ΔFT4	0.16 (-0.07 to 0.38)	.17	0.15 (-0.09 to 0.37)	.22
**FT3 (pg/mL)**				
Baseline	-0.33 (-0.53 to -0.11)	.00	-0.34 (-0.54 to -0.12)	.00
32w	-0.20 (-0.41 to 0.04)	.10	-0.21 (-0.43 to 0.03)	.08
4 weeks after the 3rd dose	-0.26 (-0.47 to -0.03)	.03	-0.29 (-0.49 to -0.05)	.02
ΔFT3	0.10 (-0.13 to 0.33)	.39	0.09 (-0.15 to 0.31)	.48

Sex was recorded as 0: male and 1: female. The other dichotomous variables were recorded as 0: no/absent and 1: yes/present. N/A, Not applicable.

### Response of TgAb and TPOAb by the third dose of SARS-CoV-2 BNT162b2 mRNA vaccine

Clinical characteristics of the 33 participants, whose serum samples were analyzed for TgAb and TPOAb, are shown in **Table 3**. The mean TgAb was increased from 140 (SD, 459) IU/mL 32 weeks after the second dose (pre-third dose) to 174 (SD, 626 IU/mL) 4 weeks after the third dose (post-third dose) (*P* = 0.003). The mean TPOAb was not significantly changed between those two time points [mean (SD); 48 (128) vs. 51 (133) (IU/mL), *P* = 0.08]. To investigate the predictive factors for the increase of TgAb, ΔTgAb was calculated as described in the Materials and Methods section, similarly to TRAb. By univariate analysis, ΔTgAb was positively correlated with the history of thyroid disease (β = 0.553, *P* = 0.0008) and TRAb (β = 0.384, *P* = 0.03), TgAb (β = 0.975, *P <*0.0001) and TPOAb (β = 0.763, *P <*0.0001) 32 weeks after the second dose (pre-third dose) (**Table 4**). TgAb 4 weeks after the third dose was also associated with history of thyroid disease (β = 0.605, *P* = 0.0002) (**Table 4**).

**Table 3:** Baseline cohort characteristics for TgAb and TPOAb analysis (n = 33).

Characteristics	Value
Age (y), median (IQR)	49 (41-51)
Sex (Female), n (%)	20 (61)
Body mass index (kg/m^2^), median (IQR)	21 (20-23)
Smoking, n (%)	8 (24)
Alcohol (g/week), median (IQR)	0 (0-32)
Fever, n (%)	10 (30)
History of thyroid disease, n (%)	3 (9)
Family history of thyroid disease, n (%)	1 (3)
Comorbidities	
Asthma, n (%)	2 (6)
Hypertension, n (%)	5 (15)
Dyslipidemia, n (%)	2 (6)
Malignancy, n (%)	2 (6)
Diabetes mellitus, n (%)	0 (0)
Autoimmune disease, n (%)	1 (3)
Cerebral infarction, n (%)	0 (0)
Current medication	
Allergy, n (%)	4 (12)
Hypertension, n (%)	4 (12)
Dyslipidemia, n (%)	2 (6)
Diabetes mellitus, n (%)	0 (0)
Immunosuppressant, n (%)	1 (3)
Anti-SARS-CoV-2 S IgG antibody	
Baseline IgG titer (U/mL), median (IQR)	<0.4 (<0.4-<0.4)
4 weeks after vaccination	
Days after 2nd dose, median (IQR)	28 (28-28)
IgG titer (U/mL), median (IQR)	923 (642-1377)
24 weeks after vaccination	
Days after 2nd dose, median (IQR)	168 (168-168)
IgG titer (U/mL), median (IQR)	399 (233-682)
32 weeks after vaccination	
Days after 2nd dose, median (IQR)	224 (224-224)
IgG titer (U/mL), median (IQR)	316 (176-538)
4 weeks after 3rd vaccination	
Days after 3rd dose, median (IQR)	28 (28-28)
IgG titer (U/mL), median (IQR)	16885 (10566-21842)
TRAb (IU/L)	
Baseline, mean (SD)	0.82 (0.07)
32w, mean (SD)	0.91 (0.14)
4 weeks after the 3rd dose, mean (SD)	1.07 (0.34)
TSH (μIU/mL)	
Baseline, mean (SD)	2.02 (1.94)
32w, mean (SD)	1.91 (1.52)
4 weeks after the 3rd dose, mean (SD)	1.71 (1.32)
FT4 (ng/mL)	
Baseline, mean (SD)	1.20 (0.12)
32w, mean (SD)	1.20 (0.15)
4 weeks after the 3rd dose, mean (SD)	1.18 (0.16)
FT3 (pg/mL)	
Baseline, mean (SD)	2.94 (0.39)
32w, mean (SD)	3.02 (0.35)
4 weeks after the 3rd dose, mean (SD)	2.87 (0.39)
TgAb (IU/mL)	
32w, mean (SD)	140 (459)
4 weeks after the 3rd dose, mean (SD)	174 (626)
TPOAb (IU/mL)	
32w, mean (SD)	48 (128)
4 weeks after the 3rd dose, mean (SD)	51 (133)

IQR, interquartile range. Sex was recorded as 0: male and 1: female.

**Table 4:** Univariate analysis for increases of TgAb (ΔTgAb) and TgAb 4 weeks after the third dose vaccine (n = 33).

	ΔTgAb		TgAb (4 weeks after the third dose)	
Variable	β (95% CI)	*P value*	β (95% CI)	*P value*
Age (y)	0.08 (-0.27 to 0.41)	.67	0.11 (-0.25 to 0.43)	.56
Sex	0.16 (-0.19 to 0.48)	.38	0.20 (-0.16 to 0.51)	.27
Body mass index (kg/m^2^)	0.12 (-0.24 to 0.44)	.52	0.11 (-0.24 to 0.43)	.55
Smoking	-0.11 (-0.44 to 0.24)	.55	-0.10 (-0.43 to 0.25)	.58
Alcohol (g/week)	-0.11 (-0.44 to 0.24)	.55	-0.14 (-0.46 to 0.22)	.45
Fever	-0.10 (-0.43 to 0.25)	.57	-0.08 (-0.42 to 0.27)	.64
History of thyroid disease	0.55 (0.26 to 0.75)	.00	0.61 (0.33 to 0.79)	.00
Family history of thyroid disease	-0.03 (-0.37 to 0.31)	.85	-0.04 (-0.38 to 0.30)	.80
Anti-SARS-CoV-2 S Ab IgG titer (U/mL)				
Pre-vaccination (Baseline)	N/A		N/A	
4 weeks after the 2nd dose	-0.05 (-0.39 to 0.30)	.78	0.03 (-0.32 to 0.37)	.87
24 weeks after the 2nd dose	-0.02 (-0.36 to 0.32)	.90	0.04 (-0.31 to 0.38)	.84
32 weeks after the 2nd dose	0.07 (-0.28 to 0.40)	.69	0.15 (-0.21 to 0.47)	.42
4 weeks after the 3rd dose	0.02 (-0.32 to 0.36)	.90	0.08 (-0.28 to 0.41)	.68
TRAb (IU/L)				
Baseline	-0.04 (-0.38 to 0.31)	.82	-0.06 (-0.39 to 0.29)	.76
32w	0.38 (0.05 to 0.64)	.03	0.38 (0.04 to 0.64)	.03
4 weeks after the 3rd dose	0.12 (-0.23 to 0.45)	.49	0.11 (-0.25 to 0.43)	.55
ΔTRAb	0.14 (-0.21 to 0.46)	.44	0.13 (-0.23 to 0.45)	.48
TSH (μIU/mL)				
Baseline	0.00 (-0.35 to 0.34)	.99	-0.01 (-0.35 to 0.34)	.97
32w	-0.06 (-0.39 to 0.29)	.76	-0.06 (-0.39 to 0.29)	.75
4 weeks after the 3rd dose	-0.09 (-0.42 to 0.26)	.62	-0.09 (-0.42 to 0.26)	.63
ΔTSH	-0.09 (-0.42 to 0.26)	.63	-0.08 (-0.41 to 0.27)	.66
FT4 (ng/dL)				
Baseline	-0.23 (-0.53 to 0.13)	.21	-0.22 (-0.52 to 0.14)	.22
32w	-0.30 (-0.59 to 0.05)	.09	-0.29 (-0.58 to 0.06)	.10
4 weeks after the 3rd dose	-0.27 (-0.56 to 0.08)	.12	-0.27 (-0.56 to 0.09)	.14
ΔFT4	-0.14 (-0.46 to 0.21)	.43	-0.14 (-0.46 to 0.21)	.43
FT3 (pg/mL)				
Baseline	-0.12 (-0.44 to 0.23)	.51	-0.17 (-0.49 to 0.18)	.34
32w	-0.12 (-0.44 to 0.24)	.52	-0.14 (-0.46 to 0.22)	.45
4 weeks after the 3rd dose	-0.19 (-0.50 to 0.17)	.30	-0.23 (-0.53 to 0.13)	.21
ΔFT3	-0.11 (-0.43 to 0.25)	.56	-0.08 (-0.42 to 0.27)	.64
TgAb (IU/mL)				
32w	0.98 (0.95 to 0.99)	.00	1.00 (1.00 to 1.00)	.00
4 weeks after the 3rd dose	0.99 (0.97 to 0.99)	.00	N/A	
TPOAb (IU/mL)				
32w	0.76 (0.57 to 0.88)	.00	0.76 (0.56 to 0.87)	.00
4 weeks after the 3rd dose	0.73 (0.52 to 0.86)	.00	0.73 (0.51 to 0.86)	.00

Sex was recorded as 0: male and 1: female. The other dichotomous variables were recorded as 0: no/absent and 1: yes/present. N/A, Not applicable.

## Discussion

In this twelve-month follow-up study in a cohort of Japanese healthcare workers, we showed the first evidence of the SARS-CoV-2 BNT162b2 vaccine increasing TRAb, as a maker of thyroid autoimmunity. In addition, we presented candidate predictive factors to increase TRAb by the vaccine: female sex, baseline low FT4 and FT3. Furthermore, as confirmation of the disruption of the thyroid autoimmunity by the vaccine, we demonstrated that the third dose of mRNA vaccine increased TgAb in 4 weeks.

One of the main findings of this study is that TRAb was significantly increased by the first two doses of the SARS-CoV-2 BNT162b2 vaccine and that the effect was still evident at 32 weeks. Furthermore, although without statistical significance, TRAb tended to be additionally increased by the third dose of the vaccine within 4 weeks. Since most of the participants (67/70) had baseline TRAb under the lower limit of the reference range (<0.8 IU/L), it is assumed that the SARS-CoV-2 BNT162b2 vaccine not only disrupted the steady state of GD, but also newly induced the disruption of thyroid autoimmunity. These findings support previous cases and series that showed the relapse or even new onset of GD potentially associated with the SARS-CoV-2 BNT162b2 mRNA vaccine (3, 7, 10). Similar to TRAb, TgAb was also increased after the third dose, additionally supporting the evidence about the disruption of thyroid autoimmune owning to the vaccine. Although all the participants received the BNT162b2 mRNA vaccine in this study, since GD was also newly induced or enhanced by other SARS-CoV-2 mRNA vaccines in previous case reports, the effects may be considered as a class effect of the drug (3, 7, 10, 11). Supportively, a recent study in patients with GD also showed an increasing trend of TRAb from one month after to three months after the administration of inactivated SARS-CoV-2 vaccines (12). Together, our data with the results of previous case reports provide evidence that the SARS-CoV-2 mRNA vaccine disrupts thyroid autoimmunity.

In this study, no participants had new onset of GD with clinically overt hyperthyroidism. The criteria for GD was immunologically fulfilled in 4.3% of the participants (3/70) with the positivity of TRAb (TRAb>2), but none had overt hyperthyroidism. Conversely, although not significantly, TSH was decreased in a time-dependent manner, suggesting the possibility of progression in subclinical hyperthyroidism after the vaccine. Supportively, the responders to increase in TRAb showed less increase of TSH and more increase of FT4 than non-responders (Table S1). Conversely, FT3, and to a lesser extent FT4, was tended to be decreased after the third vaccine in the overall subjects. This might be because of non-thyroidal illness or disturbance of FT4 to FT3 conversion following the mRNA vaccination in non-responders. Further clinical evidence from studies with larger samples is required to clarify if the mRNA vaccine could have the potential to induce GD.

The mechanisms of how the SARS-CoV-2 mRNA vaccine developed thyroid autoimmunity is still debatable. Besides GD and subacute thyroiditis, silent thyroiditis, concurrent GD and SAT, and painless thyroiditis have also been reported as COVID-19 vaccination-related thyroid diseases (4, 13). Two possible mechanisms that have been mainly discussed to date are the induction of the autoimmune/inflammatory syndrome induced by adjuvants (ASIA), and molecular mimicry of the SARS-CoV-2 mRNA vaccines encoding proteins that may cross-react with thyroid antigens (3, 14–18). ASIA is induced by adjuvants and develops through disruption of the host’s immunological balance in genetically-susceptible subjects, involving post-vaccination autoimmune phenomena. Adjuvants are included in vaccines to enhance immune responses specifically against pathogens. However, some adjuvants can trigger adverse immune reactions, as evidenced in animal models (15, 16). The RNA-based vaccines are delivered to the host cells *via* adjuvants including lipid nanoparticles (19). Exposure to the adjuvants can potentially induce an exaggerated immune response (20) and precipitate the development of thyroid autoimmunity (3, 14, 17, 21). A review article of 83 reported cases of patients that received various types of COVID-19 vaccines, including mRNA-based, viral vector-based, and inactivated vaccines, revealed that the most cases of thyroid abnormalities following the vaccinations were observed after mRNA-based vaccines (4). The mechanisms are still unknown, but one of the reasons may be because mRNA itself acted as an adjuvant due to its intrinsic immunostimulatory properties (22). Furthermore, since the intrinsic adjuvant activity of the vaccines can trigger the innate sensors, the study to evaluate the associations between production by innate sensors, such as cytokines and thyroid function or thyroid autoimmune antibodies, would be expected to further support the effect of ASIA on thyroid autoimmunity (22).

As for the molecular mimicry of the mRNA vaccines, SARS-CoV-2 spike protein is known to cross-react with thyroid peroxidase (18). This may explain the discrepancy of the induction between TgAb and TPOAb following the mRNA vaccine, suggesting the predominant possibility of ASIA rather than molecular mimicry. As for TRAb, molecular similarity of SARS-CoV-2 spike protein and TSH receptor remains unclear, but the expression of TSH receptor has been reported to be increased during SARS-CoV-2 infection (23). We therefore speculate that both TSH receptor and SARS-CoV-2 spike protein epitope could be bound and presented by antigen-presentation cells in disease-susceptible hosts, as was reported in patients with GD (24–26). In addition, the cross-reactivity of SARS-CoV-2 with thyroid target proteins may also facilitate the triggering of ASIA syndrome. Conversely, considering incidences of the other autoimmune antibody-related diseases possibly due to the SARS-CoV-2 mRNA vaccine, such as type 1 diabetes or isolated adrenocorticotropic hormone deficiency, there may be production of new autoimmune antibodies against target endocrine organs (8, 21, 27, 28). Further studies to elucidate how the mRNA vaccine disrupts the thyroid autoimmunity are anticipated.

Another feature of this study is that we presented the factors associated with the increase of TRAb (ΔTRAb) and TgAb (ΔTgAb) after the vaccine, which could be possible predictive factors for future development of thyroid autoimmunity. Female sex and low FT4 and FT3 at baseline were candidate predictive factors for the increase of TRAb by the vaccine. The factor of female sex may suggest that those with the clinical background of autoimmune thyroid disease are prone to be affected by the vaccine. Similarly, low FT4 and FT3 at baseline associated with ΔTRAb may suggest pre-existing thyroid autoimmunity to induce hypothyroidism, such as Hashimoto’s thyroiditis undetected at the initial interview. On the other hand, past history of thyroid disease and TRAb, TgAb and TPOAb before the third vaccine were candidates for the increase of TgAb after the third vaccine. These findings further suggest that thyroid autoimmune disease-prone clinical backgrounds may play an important role in disruption of thyroid autoimmunity triggered by the vaccine. Clinically, since some of these factors (sex and history of thyroid diseases) were ascertained by simple interview, this knowledge might be useful for the endocrinologists and primary care physicians in prediction of future progression or new onset of autoimmune thyroid disease at the timing of the additional booster shots.

Regarding the interpretation and the limitations of this study, it should be noted that the majority of the participants were female, and the sample size was comparatively small. Overt autoimmune thyroid disease was not discovered in this vaccination study with the limited sample size. Nonetheless, an increase in TRAb, which was in the absence of disease manifestations, may be due to a bystander activating effect of certain B-cell clones which potentially contributes to the development of GD. On the other hand, the evaluation of thyroid autoimmunity by TgAb and TPOAb was performed in limited subjects and over a relatively short time periods, and this could be a cause of discrepancies in the significant increase in between TRAb and TgAb/TPOAb. Careful interpretation is required if the effect of current study is specific to SARS-CoV-2 mRNA vaccine. Setting a control group of participants who had received another vaccine such as seasonal flu shot would be helpful in future studies to clarify the specific effect of SARS-CoV-2 mRNA vaccine.

Other limitations of this study include the lack of measurement of cellular immunity and neutralizing antibody titers against SARS-CoV-2 and morphological assessment of thyroid with ultrasonography. In this study, there was no association between SARS-CoV-2 IgG antibody and TRAb. Despite evidence of a correlation between IgG response and protection against COVID-19, cellular immunity or neutralizing antibody titers might provide a more accurate association with thyroid autoimmunity (29, 30).

In conclusion, this is the first study to demonstrate the induction of TRAb after the SARS-CoV-2 BNT162b2 mRNA vaccine. Furthermore, this study presented several candidate factors to predict the increase of TRAb after the vaccine. In addition, this study also presented the increase of TgAb after the third dose. These findings support the increasing number of previous case reports regarding disruption of thyroid autoimmunity. There is abundant beneficial evidence of the mRNA vaccine against the severe outcomes of hospitalization and death. However, early identification of relapse or even new-onset of GD should also be considered to facilitate prompt diagnosis and initiation of treatment. Further epidemiological evidence with a larger cohort within mechanistic studies is required to confirm the associations between the SARS-CoV-2 mRNA vaccines and the development of thyroid autoimmunity.

## Data availability statement

The original contributions presented in the study are included in the article/**Supplementary Material**. Further inquiries can be directed to the corresponding authors.

## Ethics statement

The studies involving human participants were reviewed and approved by Wakayama City Medical Association Seijinbyo Center. The patients/participants provided their written informed consent to participate in this study.

## Author contributions

SM and TT had full access to all of the data in the study and take responsibility for the integrity of the data and the accuracy of the data analysis. SM and TT contributed equally to this work. Concept and design: SM and TT. Acquisition, analysis, or interpretation of data: all authors. Drafting of the manuscript: all authors. Critical revision of the manuscript for important intellectual content: SM and TT. Statistical analysis: SM and TT. Obtained funding: not applicable. Administrative, technical, or material support: SM and TT. Supervision: SM and TT. Competing interests: All the authors contributed to the article and approved the submitted version.
