# Erratum: Effect of SARS-CoV-2 BNT162b2 mRNA vaccine on thyroid autoimmunity: a twelve-month follow-up study

**DOI:** 10.3389/fendo.2023.1257424

**Published:** 2023-07-24

**Authors:** Frontiers Production Office

**Affiliations:** Frontiers Media SA, Lausanne, Switzerland

**Keywords:** SARS-CoV-2, COVID-19, thyroid autoimmunity, BNT162b2 vaccine, thyroid-stimulating hormone receptor antibody, Graves’ disease

Due to a production error, an author’s name was incorrectly spelled as “Taka-aki Mastuoka”. The correct spelling is “Taka-aki Matsuoka”. The publisher apologizes for this mistake.

The original version of this article has been updated.

