# Supplementary figures and images for: Effect of SARS-CoV-2 BNT162b2 mRNA vaccine on thyroid autoimmunity: A twelve-month follow-up study
Source: Front Endocrinol (Lausanne). 2023 Jan 27;14:1058007. doi: 10.3389/fendo.2023.1058007 (PMC9911871; doi:10.3389/fendo.2023.1058007)

Figure S1. Flow diagram of the participants

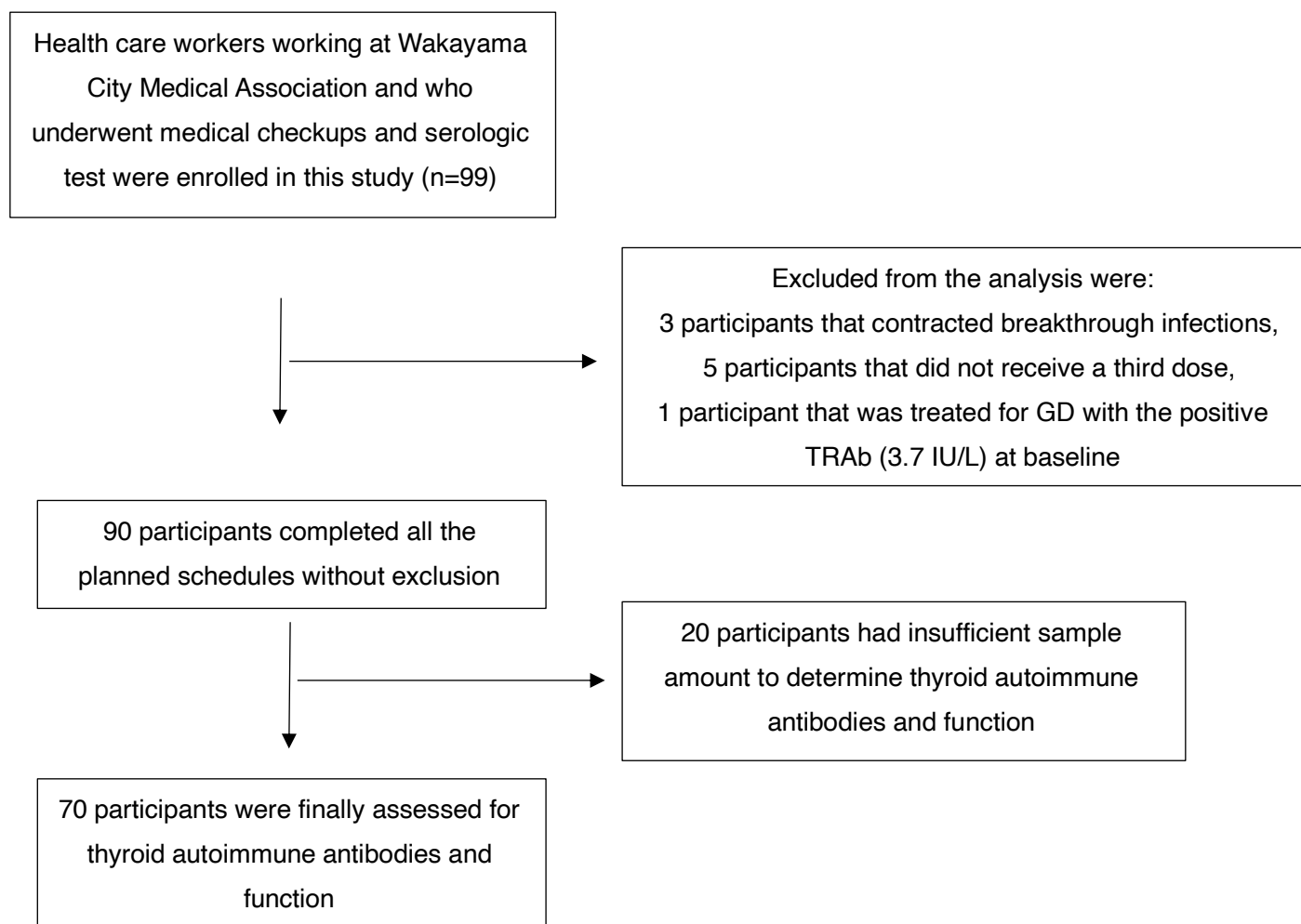

Supplement: Supplementary file 1 [file DataSheet_1.pdf]
