# Supplementary material for: Effect of SARS-CoV-2 BNT162b2 mRNA vaccine on thyroid autoimmunity: A twelve-month follow-up study
Source: Front Endocrinol (Lausanne). 2023 Jan 27;14:1058007. doi: 10.3389/fendo.2023.1058007 (PMC9911871; doi:10.3389/fendo.2023.1058007)
Supplement: Supplementary file 2 [file DataSheet_2.docx]

Table S1. Clinical differences between responder and non-responder to increase TRAb

|  |  | Responder (n=12) |  | Non-responder　(n=58) | *P value* |
| --- | --- | --- | --- | --- | --- |
| ΔTRAb, mean (SD) |  | 0.63 (0.35) |  | 0.06 (0.01) | .00 |
| ΔTSH, mean (SD) |  | -1.10 (2.50) |  | -0.18 (0.12) | .02 |
| ΔFT4, mean (SD) |  | 0.12 (0.26) |  | -0.04 (0.01) | .01 |
| ΔFT3, mean (SD) |  | 0.12 (0.37) |  | -0.09 (0.03) | .10 |
|  |  |  |  |  |  |
| Age (y), median (IQR) |  | 45 (36-50) |  | 50 (39-55) | .09 |
| Sex (Female), n (%) |  | 12 (100) |  | 36 (62) | .01 |
| Body mass index (kg/m^2^), median (IQR) |  | 22 (20-23) |  | 22 (20-24) | .68 |
| Smoking, n (%) |  | 2 (17) |  | 12 (21) | .99 |
| Alcohol (g/week), median (IQR) |  | 0 (0-18) |  | 0 (0-54) | .84 |
| Fever, n (%) |  | 6 (50) |  | 17 (29) | .19 |
|  |  |  |  |  |  |
| History of thyroid disease, n (%) |  | 3 (25) |  | 1 (2) | .01 |
| Family history of thyroid disease, n (%) |  | 0 (0) |  | 3 (5) | .64 |

IQR, interquartile range

Sex was recorded as 0: male and 1: female. The other dichotomous variables were recorded as 0: no/absent and 1: yes/present.

Responders were defined as described in the Materials and Methods section.
